# Thoracic Branching Endoprosthesis for Management of Coarctation of the Aorta With Subclavian Artery Involvement

**DOI:** 10.1016/j.jscai.2024.101335

**Published:** 2024-02-20

**Authors:** Pradyumna Agasthi, Bernardo C. Mendes, Allison K. Cabalka, Randall R. DeMartino, Gabor Bagameri, Alexander C. Egbe, William R. Miranda, Jason H. Anderson

**Affiliations:** a Division of Structural Heart Diseases, Department of Cardiovascular Medicine, Mayo Clinic, Rochester, Minnesota; b Division of Vascular and Endovascular Surgery, Department of Surgery, Mayo Clinic, Rochester, Minnesota; c Division of Pediatric Cardiology, Department of Pediatric and Adolescent Medicine, Mayo Clinic, Rochester, Minnesota; d Department of Cardiovascular Surgery, Mayo Clinic, Rochester, Minnesota

**Keywords:** coarctation of aorta, endovascular aortic repair, stent graft

Management of coarctation of the aorta (CoA) in adults comprise both surgical and transcatheter stent-based therapies with comparable short-term and midterm outcome data.^1^ Stent-based therapy allows for a quick recovery, minimal postoperative hospitalization, and early return to work. The primary limitation to candidacy is often the proximity of the brachiocephalic vessels to the affected area. If concern for aortic wall disruption is present, then a covered stent will often be preferred, but placement over a brachiocephalic origin will result in occlusion. Thus, if CoA is in immediate apposition to a brachiocephalic vessel, therapeutic options are primarily limited to use of an uncovered stent, surgical revascularization (carotid-subclavian bypass) before covered stent placement, or referral for surgical aortic arch reconstruction.

An endovascular product with a purpose-built side-branch could offer an additional therapeutic option to this population. The GORE TAG thoracic branch endoprosthesis (TBE; W.L. Gore) is a US Food and Drug Administration-approved graft designed for endovascular repair of pathologies of the descending thoracic aorta requiring a proximal landing zone, involving the subclavian artery in patients at high risk of debranching subclavian procedures.^2^ It is designed for transcatheter implantation with multiple graft and side-branch sizes available. Traditionally, it is considered a therapeutic option for aneurysm, dissection, and transsection but may serve a role for treatment of congenital heart disease.

**Figure 1:**
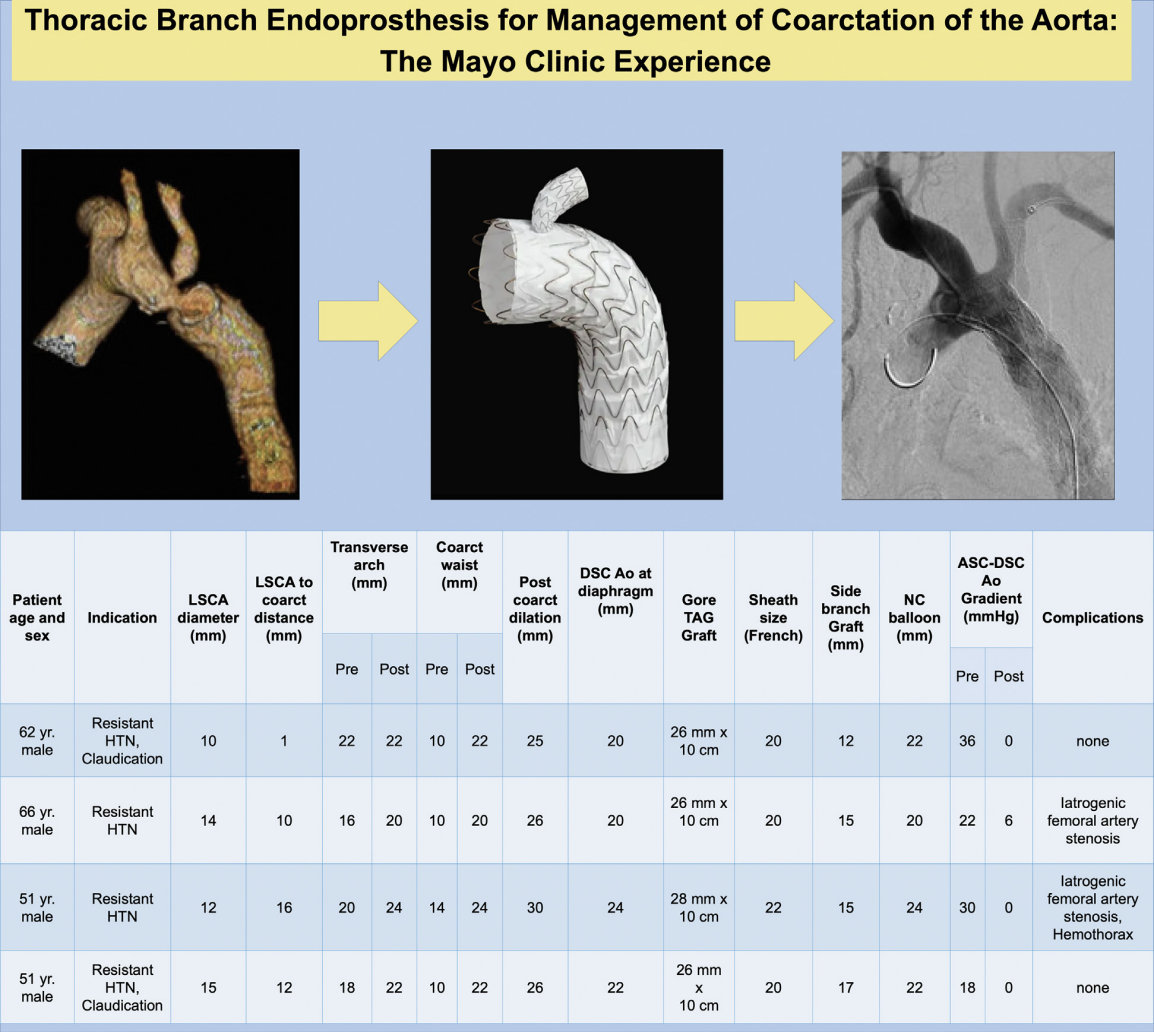
**Demographic andproceduraldata for a series of patients who underwent placement of a thoracic branch endoprosthesis for the management of coarctation of the aorta**. All patients had coarctation of aortic with prior surgical repair with end-end anastomosis. Ao, aorta; ASC, ascending; DSC, descending; HTN, hypertension; LSCA, left subclavian artery; NC, non-compliant.

We report novel utilization of this device for treatment of re-CoA in adults. Four men with a median age of 56.5 years (IQR, 13 years) underwent the procedure. All patients underwent previous surgical repair of CoA with end-to-end anastomosis. Indications for the reintervention were medically refractory hypertension (4/4 patients) and claudication (2/4 patients). The median preprocedural CoA mean systolic Doppler gradient was 26 mm Hg (IQR, 13 mm Hg). One patient had a history of Shone complex with mechanical mitral and aortic valve replacements. The median left subclavian to coarctation segment distance, coarctation diameter, and transverse arch diameters were 12.5 mm (IQR, 10.0 mm), 10.0 mm (IQR, 2.0 mm), and 19.0 mm (IQR, 4.0 mm), respectively. All procedures were performed successfully (Figure 1).

A preoperative computed tomography angiography of the thoracic aorta was used for graft selection. The device was selected in accordance with manufacturer’s guidelines with a wide range of sizes available. Sizing included both the graft body diameter (21.0-45.0 mm) and length (10.0-15.0 mm) in addition to side-branch distal diameter (8.0-20.0 mm). Selection of the graft body was based on the diameter of unaltered aorta proximal and distal to the intended treatment site, with selection targeting the largest diameter of these dimensions, because the graft functions well in the setting of oversizing. Graft length was selected to ensure coverage of aneurysmal tissue distal to the coarctation segment. A distance from the midorigin of the left common carotid to distal origin of the left subclavian artery (minimum 2.0 cm for grafts of <37.0 mm diameter) was confirmed. The vertebral artery origin was identified with a distance of >3.0 cm from the left subclavian artery, confirmed to ensure no graft impingement. Then, side-branch diameter was selected to the diameter of normal distal subclavian artery tissue beyond any proximal aneurysm.

The procedural approach comprised right radial artery access for pigtail placement in the ascending aorta to allow for angiographic guidance during the procedure. Left radial access was used to facilitate a left radial to femoral sheath wire rail using a 0.025-inch × 480.0-cm Tracer Metro Direct wire (Cook Medical). Femoral arterial access was used for placement of a DrySeal sheath (W.L. Gore) to facilitate delivery of the TBE. The Metro Direct wire was snared and externalized through DrySeal sheath. The thoracic endoprosthesis has a port for the 0.035-inch wire and 0.025-inch wire. The TBE was advanced over both the wires. Rapid ventricular pacing with a pacing catheter in the right ventricle was performed in 3 of the 4 patients. Lunderquist wire placement in the ascending aorta served as the primary interventional wire to ensure proper implantation of the TBE body. High-pressure balloon angioplasty of the graft was performed after thoracic graft implantation to facilitate planned vessel wall disruption and resolve the coarctation. The side-branch endoprosthesis was advanced over the femoral-subclavian wire rail (0.025-inch wire) into left subclavian artery through the preexisting port in the main thoracic endograft prosthesis. After deployment, the graft was dilated with a low-pressure balloon to ensure full expansion.

There was no residual ascending to descending thoracic aorta gradient with increase in transverse arch diameter in 3 of the 4 patients (Figure 1). After TBE implantation, there was no difference in the diameter of the coarctation segment and descending thoracic aorta at the level of the diaphragm. Two patients were treated with traditional closure devices, and 2 underwent surgical right femoral artery cut downs. One patient developed left hemothorax after the graft and angioplasty consistent with a dissection involving the subclavian artery with resolution after side-branch placement. This represented the most-feared complication of stent-based therapy for this population, which was managed successfully after TBE side-branch implantation. Empiric spinal drain placement was not pursued for thoracic endovascular graft implantation in our practice. Postoperative neurologic abnormalities were managed through spinal drain placement but this was not required for the cases described in this study.

To our knowledge, this is the first utilization of TBE for management of recurrent CoA with challenging anatomy that would have previously been referred for surgical repair or revascularization based on location with involvement of the left subclavian artery. An additional benefit of the TBE product is that extenders are readily available for both proximal and distal utilization should a dissection propagate beyond the covered segment. Current stent-based therapy would require utilization of a second stent, which can be quite difficult to perform once resolution of the waist has occurred because there may be no waist to seat the stent. Alternatively, the patient may require emergent conversion to surgical repair.

Important lessons extrapolated from our initial experience include the following:

1. Routine use of ventricular pacing is beneficial. There is marginal benefit during graft implantation but significant benefit during high-pressure balloon angioplasty. In the absence of rapid pacing, ventricular systole during balloon dilation can result in distal migration of the TBE.
2. Minimize the time lag between high-pressure angioplasty and side-branch graft implantation because disruption of coarct segment can extend into the subclavian artery causing hemothorax. We now perform balloon angioplasty with the undeployed side-branch graft in place in the left subclavian to facilitate rapid deployment.
3. Large bore access may require vascular repair, especially in the setting of peripheral vascular disease, and thus, collaboration between interventional cardiology and vascular surgery is essential.

An interesting observation is that peripheral systolic blood pressure augmentation, which, although traditionally not observed after balloon-expandable covered stent placement, was observed after this procedure in these patients, presumed secondary to the distensibility of the graft, as opposed to a rigid stent frame. This may be more in line with native physiologic flow patterns when compared with those in traditionally stent placement. Overall, we believe this approach offers improved safety regarding purposeful aortic disruption for treatment of recurrent CoA. This series demonstrates the feasibility of TBE utilization for CoA, and a multidisciplinary collaboration to the care of these patients is essential.
